# ‘Seeing’ the electromagnetic spectrum: spotlight on the cryptochrome photocycle

**DOI:** 10.3389/fpls.2024.1340304

**Published:** 2024-03-01

**Authors:** Blanche Aguida, Jonathan Babo, Soria Baouz, Nathalie Jourdan, Maria Procopio, Mohamed A. El-Esawi, Dorothy Engle, Stephen Mills, Stephan Wenkel, Alexander Huck, Kirstine Berg-Sørensen, Sotirios C. Kampranis, Justin Link, Margaret Ahmad

**Affiliations:** ^1^Unite Mixed de Recherche (UMR) Centre Nationale de la Recherche Scientifique (CNRS) 8256 (B2A), Institut de Biologie Paris-Seine (IBPS), Sorbonne Université, Paris, France; ^2^Department of Biophysics, Faculty of Arts and Sciences, Johns Hopkins University, Baltimore, MD, United States; ^3^Botany Department, Faculty of Science, Tanta University, Tanta, Egypt; ^4^Biology Department, Xavier University, Cincinnati, OH, United States; ^5^Chemistry Department, Xavier University, Cincinnati, OH, United States; ^6^Umeå Plant Science Centre, Department of Plant Physiology, Umeå University, Umeå, Sweden; ^7^DTU Physics, Technical University of Denmark, Kongens Lyngby, Denmark; ^8^DTU Health Technology, Technical University of Denmark, Kongens Lyngby, Denmark; ^9^Biochemical Engineering Group, Plant Biochemistry Section, Department of Plant and Environment Sciences, University of Copenhagen, Frederiksberg, Denmark; ^10^Physics and Engineering Department, Cincinnati, OH, United States

**Keywords:** cryptochrome, photoreceptor, flavoprotein, redox, photomorphogenesis, circadian clock, magnetic fields, ROS

## Abstract

Cryptochromes are widely dispersed flavoprotein photoreceptors that regulate numerous developmental responses to light in plants, as well as to stress and entrainment of the circadian clock in animals and humans. All cryptochromes are closely related to an ancient family of light-absorbing flavoenzymes known as photolyases, which use light as an energy source for DNA repair but themselves have no light sensing role. Here we review the means by which plant cryptochromes acquired a light sensing function. This transition involved subtle changes within the flavin binding pocket which gave rise to a visual photocycle consisting of light-inducible and dark-reversible flavin redox state transitions. In this photocycle, light first triggers flavin reduction from an initial dark-adapted resting state (FADox). The reduced state is the biologically active or ‘lit’ state, correlating with biological activity. Subsequently, the photoreduced flavin reoxidises back to the dark adapted or ‘resting’ state. Because the rate of reoxidation determines the lifetime of the signaling state, it significantly modulates biological activity. As a consequence of this redox photocycle Crys respond to both the wavelength and the intensity of light, but are in addition regulated by factors such as temperature, oxygen concentration, and cellular metabolites that alter rates of flavin reoxidation even independently of light. Mechanistically, flavin reduction is correlated with conformational change in the protein, which is thought to mediate biological activity through interaction with biological signaling partners. In addition, a second, entirely independent signaling mechanism arises from the cryptochrome photocycle in the form of reactive oxygen species (ROS). These are synthesized during flavin reoxidation, are known mediators of biotic and abiotic stress responses, and have been linked to Cry biological activity in plants and animals. Additional special properties arising from the cryptochrome photocycle include responsivity to electromagnetic fields and their applications in optogenetics. Finally, innovations in methodology such as the use of Nitrogen Vacancy (NV) diamond centers to follow cryptochrome magnetic field sensitivity *in vivo* are discussed, as well as the potential for a whole new technology of ‘magneto-genetics’ for future applications in synthetic biology and medicine.

## 1 Introduction

Cryptochromes are members of the photolyase/cryptochrome superfamily of highly conserved flavoprotein receptors which can undergo photochemical redox reactions in response to light (Ozturk, 2017; Vechtomova et al., 2021). They are found throughout the biological kingdom, from archaebacteria to man, and participate in diverse physiological responses such as plant development and in entrainment of the circadian clock in plants and animals. Since their first identification in plants (Ahmad and Cashmore, 1993), the structural and biochemical properties of isolated cryptochrome proteins from many organisms have been extensively characterized *in vitro*, including photoreactions to the picosecond time scale (see eg. Chaves et al., 2011; Zoltowski et al., 2011; Ahmad, 2016; Schroeder et al., 2018; Hore and Mouritsen, 2016; Kondoh and Terazima, 2017; Schelvis and Gindt, 2017). At the other end of the scale, the physiological and behavioral consequences of cryptochrome activation *in vivo*, both as light sensors and as signaling molecules have been extensively studied, including those involved in magnetic orientation in birds (Wiltschko et al., 2021) and those of importance to medicine, such as in clock-related pathologies and as potential therapeutic substrates in neurobiology and other diseases (eg Michael et al., 2017; Chan and Lamia, 2020; Damulewicz and Mazzotta, 2020; Lohof et al., 2022). In addition, physiological effects of cryptochromes in crop improvement (e.g. Fantini and Facella, 2020; Wang and Lin, 2020), as well as their usefulness for the creation of optogenetic tools (Seong and Lin, 2021; Huang et al., 2022), also have significant economic potential.

In this review, we focus primarily on the question of how light absorption by certain cryptochromes mediates a biological signaling response *in vivo*. This causal connection, between cryptochrome photoreactions observed *in vitro* with biological responses observed *in vivo*, is what distinguishes these Crys from classes of photoactive proteins that simply use light as an energy source (such as photolyases, or proteins of the photosynthetic apparatus). First and foremost, a light-driven chemical transformation in the receptor must be induced by light which leads to the initiation of biological signaling activity. This results in a conformational change enabling the receptor to interact with cellular signaling proteins (see eg higher plant phytochromes and phototropins as well as the animal opsins (Galvãno and Fankhauser, 2015; Shichida and Matsuyama, 2009).

A second critical feature of a functional photoreceptor is that there must be a mechanism in place to return it to the inactive state once the light signal is gone. This must furthermore occur on a time scale consistent with its biological function. Otherwise, the receptor remains permanently in the ‘lit’ state and visually blind after the initial photon absorption event; or else returns to the dark-adapted state before it has a chance to initiate biological signaling responses.

**Figure 1:**
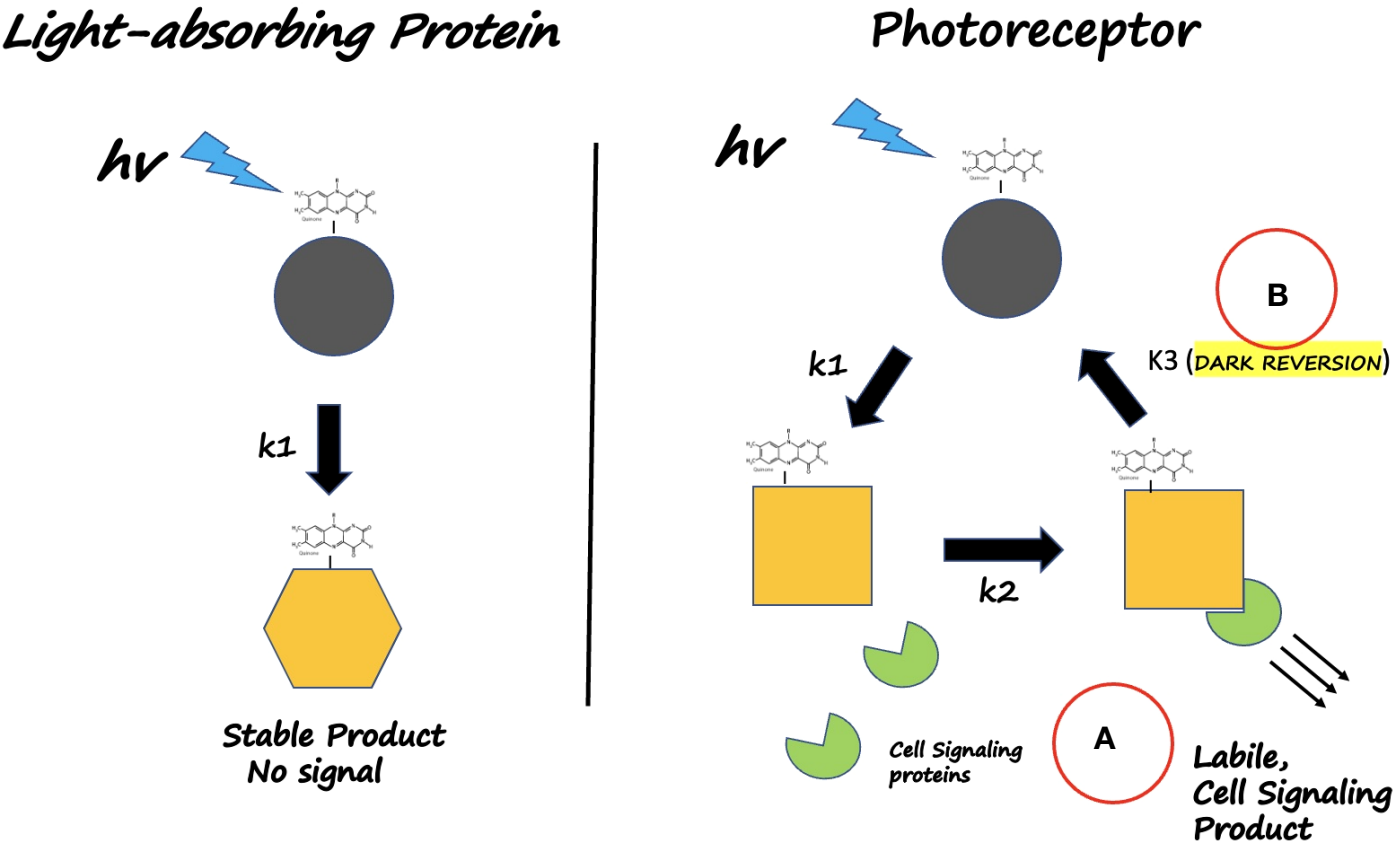
What defines a Photoreceptor? Left Panel: Light - absorbing NON- VISUAL proteins (Eg. *E*. *coli* photolyase) use light as an energy source. The dark-adapted state is partially oxidized *in vitro* and undergoes flavin reduction and conformational change (rate k1); however it does not interact with cellular signaling proteins and does not revert to the dark-adapted form on a physiological time scale. Instead, the reduced state remains stable and harvests additional light energy for catalytic repair of DNA. Right Panel: Light-absorbing Photoreceptors (plant Crys) undergo a photocycle on a time scale that reversibly triggers a physiological response. **(A)** Light absorption (rate constants k1, k2) triggers receptor conformational change that enables detection by downstream signaling proteins. **(B)** Return to darkness (rate constant k3) restores the inactive resting state on a time scale of minutes. In this way, biological activity is triggered by light but also responds to light intensity and duration (via k1, k2), relative to the rate of dark reversion (k3).

These two features comprise the so-called photocycle – the mechanism by which a biological receptor is activated by light to achieve the ‘lit’, or physiologically active, state, and then returns to the inactive ‘resting’ state once the light signal is removed. This photocycle, enables the system to respond to the light input intensity, and is outlined in generic form in **Figure 1**. In this review we will discuss the origins of the photocycle, the means by which the photocycle responds to environmental signals, and the *in vivo* physiological implications of this photocycle in the control of cryptochrome responses. Finally, we will discuss emerging topics related to novel methodology for Cry analysis using NV diamond centers, and development of novel optogenetic tools using plant Crys in yeast.

The review focusses primarily on the plant sensory Crys (Cry1 and Cry2 of *Arabidopsis*), as these are among the best characterized; however some pertinent comparisons to other cryptochromes such as avian and *Drosophila* Crys will be drawn, as well as to algal Crys.

## 2 Origins: in the beginning, there was photolyase.

At the time of the origins of life, the earth had a particularly inhospitable atmosphere consisting primarily of hydrogen sulfide, ammonia, methane, and CO_2_ (Zahnle et al., 2010). In the absence of oxygen, there was no protective ozone layer to prevent ionizing radiation from reaching the earth’s surface and the DNA-damaging effects of UVB and UVC radiation posed a major challenge to unicellular organisms. It was in this reducing, ionizing environment, around 3 billion years ago, that the first members of the DNA photolyase/cryptochrome gene family are thought to have arisen. Photolyases, or PHR, are DNA repair enzymes that use visible light to catalyse the repair of damaged DNA (Sancar, 2004; Vechtomova et al., 2021). Photolyase variants can repair each of the two principal types of DNA damage; 6-4 photoproducts and cyclobutane pyrimidine dimers (CPD). Since strongly ionizing radiation always occurred in combination with visible light on this early earth, using light as a catalyst for DNA repair was an effective survival strategy and, as a result, members of the photolyase gene family are today widely dispersed throughout the biological Kingdoms.

### 2.1 DNA repair came first

The mechanism by which photolyases use light to repair DNA is well understood. Structurally, photolyases are globular proteins that non-covalently bind two cofactors: an antenna pigment bound near the amino terminal close to the surface of the protein, and a flavin, FADH-, bound within a hydrophobic cavity at the C-terminal alpha-helical domain (see Sancar, 2004; Sancar, 2016; Zhang et al., 2017; Vechtomova et al., 2021). Photolyases recognize structural changes in DNA caused by CPD or 6-4 photoproduct damage lesions and bind to them via ionic interactions. A stable enzyme/substrate complex is then formed by flipping the damaged base pairs of the DNA into the central cavity of the enzyme proximal to the flavin. Both of these events occur in the absence of light. To initiate DNA repair, an antenna pigment, which is generally either methenyltetrahydrofolate (MTHF) or 8-hydroxy-7,8-didemethyl-5-deaza- riboflavin (8-HDF), first absorbs a photon of light and subsequently transfers this energy via FRET to the reduced flavin cofactor (FADH^-^). The resulting high energy excited state flavin (FADH*-) transfers an electron to the DNA lesion which repairs the DNA (Sancar, 2004). The electron subsequently returns to the flavin to restore the fully reduced resting state of the enzyme. This process lasts less than a microsecond and is fully catalytic.

### 2.2 ‘Photoactivation reaction’ and characterization of the ‘Trp triad’

The reaction mechanism of photolyases has important consequences on the resting redox state of the catalytic flavin cofactor. Exclusively the reduced flavin redox state (FADH^-^) of photolyases is catalytically active, as no other redox form can catalyse DNA repair. Therefore, a photolyase enzyme (either *in vitro* or *in vivo*) in which the flavin becomes artifactually oxidized loses all DNA repair function (Sancar, 2004; Brettel and Byrdin, 2010). However, purified preparations of isolated photolyases under atmospheric oxygen typically occur in various oxidized redox states and must first be reduced in order to provide functional enzymes for studies *in vitro*. This requirement for reduced flavin led researchers to discover and characterize the process of ‘photoactivation’, in which the flavin can be photoreduced into the catalytically active form.

As an example, in the case of *E.Coli* photolyase, blue light illumination of the catalytic flavin in the oxidized redox state will result in formation of the excited state flavin (FAD*), which will then extract a nearby electron from a conserved W residue (W382) within the protein. This is followed by further electron transfer from W382 to a suitably positioned conserved W359 residue, which in turn extracts an electron from surface-exposed W306. This process of intraprotein electron transfer is accompanied by the transient formation of the FAD•- anionic radical, followed by its protonation to the neutral radical FADH• state.

To achieve the catalytically active state, a second round of illumination is required. In this round, the photon is absorbed by the neutral radical FADH• to again generate an excited state (FADH*). Subsequently, intraprotein electron transfer to the flavin (FADH* <– W382 <– W359 <– W306) will yield the fully reduced, catalytically active redox state (FADH^-^). Although variations exist among different photolyases and/or cryptochromes in the ultimate electron acceptors being used (eg. a fourth terminal Y and/or W residue), the minimal electron transfer pathway consisting of the three W residues is widely referred to as the ‘Trp triad’. It is conserved throughout the cryptochrome/photolyase gene family, and is essential for generating active photolyase (PHR) in cases where the flavin has become oxidised *in vitro* (Sancar, 2004; Brettel and Byrdin, 2010; Kavakli et al., 2017; Kavakli et al., 2019; Maestre-Reyna et al., 2022).

There are two important features of the photoactivation pathway that distinguish it from DNA repair. Firstly, unlike for DNA repair, the catalytic flavin itself can serve as an efficient photon acceptor, excluding the necessity for an antenna pigment. This is because oxidized or radical state flavin can efficiently absorb visible light. In particular, the neutral radical redox state (FADH•) absorbs in UV, blue, green and yellow light, making it responsive to all wavelengths with the exception of red light (Brettel and Byrdin, 2010; Bouly et al., 2007).

### 2.3 Photolyases absorb light for DNA repair primarily via their antenna pigment

**Figure 2:**
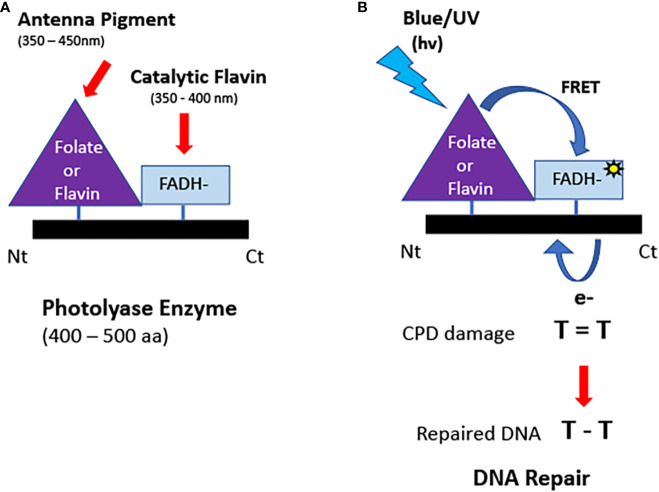
Structure and function of photolyases. **(A)** Domain structure. Photolyases typically bind two chromophores: a light-absorbing folate or deazaflavin antenna pigment and the catalytic FADH^-^ cofactor. **(B)** Enzymatic repair of DNA. Light energy is absorbed by the antennae cofactor bound to the N terminal of the PHR and transferred by FRET to generate the excited state reduced flavin. Reduced flavin transfers an electron to repair the DNA lesion (CPD or 6-4 photoproducts). The electron is restored to the enzyme following repair. The catalytically active flavin (FADH^-^) does not absorb light in the visible spectral range and therefore relies on antenna pigments to capture light energy.

The photochemical reactions undergone by photolyases harness incoming light energy for DNA repair, and are highly efficient (quantum yield of up to 0.8) (**Figure 2**). However, this process requires that the resting (dark-adapted) state of photolyase contains fully reduced flavin (FADH^-^), which does not efficiently absorb visible light (cutoff above 410nm). Instead, DNA repair is initiated primarily through photon absorption by the antenna pigment (Sancar, 2004), with peak absorption in the near-UV range (around 380nm) in the case of folate derivatives. Therefore many photolyases are essentially ‘blind’ to visible light (400 – 500nm range) and only respond efficiently to near-UV light. Exceptions occur in the case of the so-called long wavelength photolyases which repair DNA efficiently in blue light (peak near 440nm); however, these ‘long wavelength’ photolyases also absorb light primarily through an antenna pigment (deazaflavin derivatives) (Sancar, 2004). In sum, most photolyases have peak activity in the UV/A region, they function less well in the visible range, and none respond at longer wavelengths above 500nm (to turquoise, green, or red light).

### 2.4 Photolyases do not undergo a photocycle *in vivo*

One important biological requirement for efficient DNA repair is that the flavin of photolyases is stably maintained in the reduced redox state *in vivo*. The cellular resting flavin redox state is imposed by a number of parameters including protein structure of the flavin pocket in photolyase, accessibility to oxygen, and the cellular midpoint potential (Wen et al., 2022). Current evidence suggests that flavin is indeed maintained in the reduced state for photolyases *in vivo*. For example, photolyases in their purified state are notoriously difficult to oxidize, even when exposed to atmospheric conditions (oxygen concentrations orders of magnitude greater than within the cell). In the case of purified *E. coli* photolyase, full oxidation can only be achieved using added chemical oxidants (Sancar, 2004; Wen et al., 2022). This indicates that at the low cellular oxygen concentrations *in vivo* PHR is likely to remain reduced. Additional evidence that PHR is perpetually maintained in the reduced redox state comes from mutant analysis (Kavakli et al., 2017; Kavakli et al., 2019). Therefore, although it has been reported that photolyases undergo conformational changes in response to photoreduction (Cellini et al., 2022; Maestre-Reyna et al., 2022) these do not appear to be reversible *in vivo* and do not interact with the cellular signaling machinery (**Figure 2**, left panel).

In sum, photolyases are exquisitely sensitive to light but use light exclusively for a catalytic function. They do not appear to have a photosensory signaling role. They do not efficiently absorb visible light, do not interact with signaling proteins in plants, and do not undergo a redox photocycle consistent with a physiological time scale (seconds to minutes).

## 3 Cryptochromes: making the transition from photolyases to photoreceptors

A major milestone in the evolution of life occurred about 2 billion years ago when the accumulated actions of the first photosynthetic organisms resulted in the so-called ‘great oxygenation event’ giving rise to the present-day atmospheric conditions of a primarily Oxygen/Nitrogen atmosphere (Lyons et al., 2014). This event had a two-fold impact on the photolyases. First, there was less selection pressure for their DNA repair function thanks to the development of a protective ozone layer screening out the worst (>98%) of the ambient ionizing radiation (Cockell, 1998). Second, even relatively minor mutations affecting the photolyase flavin pocket now resulted in loss of enzymatic DNA repair function due to spontaneous oxidation of the catalytic flavin cofactor in an oxygenating environment. In other words, the stage was set for the development of novel functions in this widespread, ancient gene family and, indeed, phylogenetic analysis shows that the earliest likely cryptochromes, seem to have emerged around this time (Vechtomova et al., 2020).

**Figure 3:**
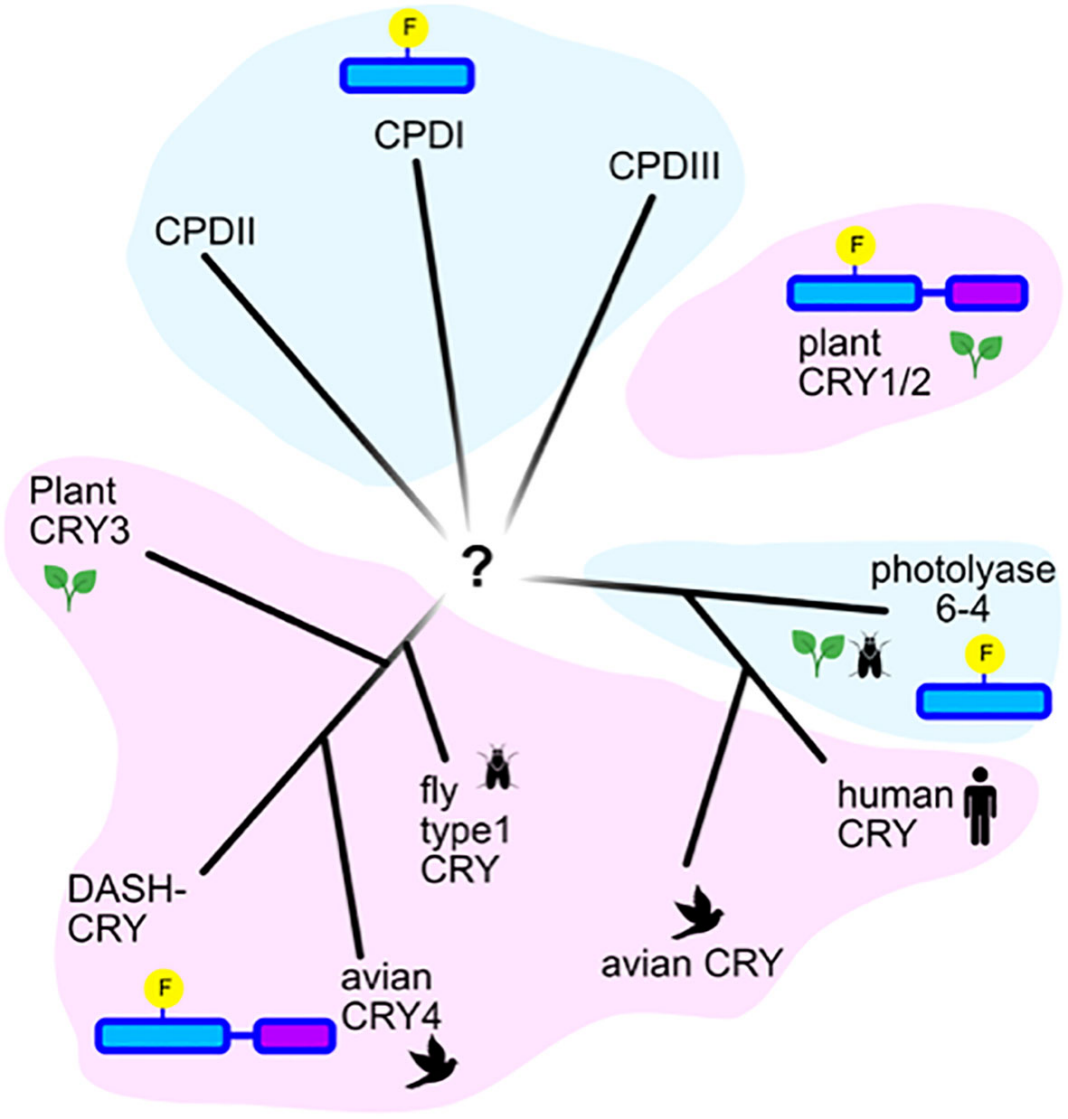
Phylogenetic relatedness of cryptochromes and photolyases. Representation of an unrooted tree showing the evolutionary relatedness of the major families of photolyases in blue shading (Types I, II, and III CPD photolyases, Type 6-4 photolyases), and of the major classes of cryptochromes (plant Cry, animal Type II Cry (human and avian), animal Type I Cry (fly), Cry-Dash, Cry4 and Cry3. According to (Ozturk, 2017).

Currently, many so-called cryptochromes have no known light signaling function, so it cannot be said that evolution of this gene family is exclusively driven by novel functional requirements. Indeed, some so-called cryptochromes designated from phylogenetic data have since been shown to retain a degree of photolyase function and, vice versa, some so-called photolyases have since been shown to have signaling roles (Vechtomova et al., 2020). Currently, there is consensus that there exist at least 6 major categories of the cryptochrome/photolyase gene family, based on evolutionary relatedness and function (**Figure 3**). These include the Type I and Type II photolyases, which are efficient in the repair of CPD DNA lesions; the Type 6-4 photolyases efficient in the repair of 6-4 photoproducts; the plant type light signaling cryptochromes Cry1 and Cry2 found in green plants and algae; the Cry-DASH cryptochrome superfamily which retain single stranded DNA repair activity; the animal Type I cryptochromes from insect; and the animal Type II cryptochromes in vertebrates and other animals including insects (For an abbreviated schematic, see **Figure 3**). This diverse array of cryptochromes results from their having evolved independently multiple times in the course of evolution, in many different organisms and from different ancestral photolyase lineages. A definition that fits most cryptochromes is that they are photolyase-like proteins with impaired dsDNA repair function, of which some have evolved novel roles in signaling.

Currently, light-sensing roles have been shown in the case of certain Cry-DASH cryptochromes (fungal and algal) (Kiontke et al., 2020), as well as cryptochrome subgroups related to 6-4 photolyases (Petersen et al., 2021). The plant-like cryptochromes related to Cry1 and Cry2, as well as the Drosophila Type I cryptochromes (Chaves et al., 2011; Deppisch et al. 2023) are among the best studied photosensory Crys.

### 3.1 Differences between plant cryptochromes and photolyases

Plant Cry1 and 2 are closely related to the Type I CPD photolyases, particularly within their N-terminal, photolyase-like domain (**Figure 3**). The most striking difference structurally between photolyases and plant type cryptochromes is the existence of a C-terminal extension of variable length (CCT). This domain, which is poorly conserved among different cryptochromes, consists of random coil sequences lacking defined structural features. The C-terminal domain is folded into the flavin binding pocket and undergoes conformational change upon light activation. In addition, it contains a nuclear localization signal as plant Cry1 and Cry2 are localized to the nucleus, although Cry1 also is found in the cytosol where it plays different developmental functions. A further structural distinction from photolyases is oligomerization of the N-terminal domain of Crys subsequent to illumination. This photo-oligomerization is necessary for Cry function and precedes interaction with downstream cellular signaling proteins (Ponnu and Hoecker, 2022). In support of their role, both oligomerisation and blue-light signaling can be perturbed by small Cry1 protein-isoforms which competitively inhibit formation of homo-oligomers (Dolde et al., 2018; Kruusvee et al. 2022). In sum, plant Crys have evolved structural modifications consistent with a signaling role.

Photochemically, plant cryptochromes undergo the same photoreduction reaction as photolyases (the so-called ‘photoactivation reaction’, see Section 2.2). This involves a sequential series of electron and proton transfer events, via the conserved Trp triad pathway similarly to E. coli photolyase, albeit with varying rate constants and quantum efficiency (see eg. Thöing et al., 2015). However, plant Crys do not catalyse light-triggered repair. This has been related to the poor conservation of the amino acid residues necessary for dsDNA binding and, even more significantly, to the fact that dark-adapted Cry flavin *in vivo* is in the fully oxidized redox state (Chaves et al., 2011; Burney et al., 2012). Thus, the flavin is not maintained in the catalytically active state required for DNA repair.

Additional differences relate to the flavin binding cavity, where plant Crys have an aspartic acid located close to the flavin which protonates the radical in the course of photoreduction (e.g. Burney et al., 2012; Müller et al., 2014). The resulting negatively charged aspartic acid renders the fully reduced state (FADH-) unstable. As a consequence, Crys occur stably in their oxidized redox state. They can absorb blue/UV-A light directly through their flavin cofactor and do not rely on antenna pigments as do photolyases. In further distinction to photolyases, Crys also bind ATP and other small molecule metabolites which stabilize the radical (FADH•) redox state (Müller and Bouly, 2014; Müller et al., 2014; Kottke et al., 2018).

In sum, although structurally very similar to photolyases, plant Crys do not catalyse DNA repair and instead undergo rapid flavin reoxidation after illumination; a C-terminal extension promotes oligomerization that contributes to signaling reactions (Müller and Ahmad, 2011; Van Wilderen et al., 2015).

### 3.2 The plant cryptochrome photocycle

**Figure 4:**
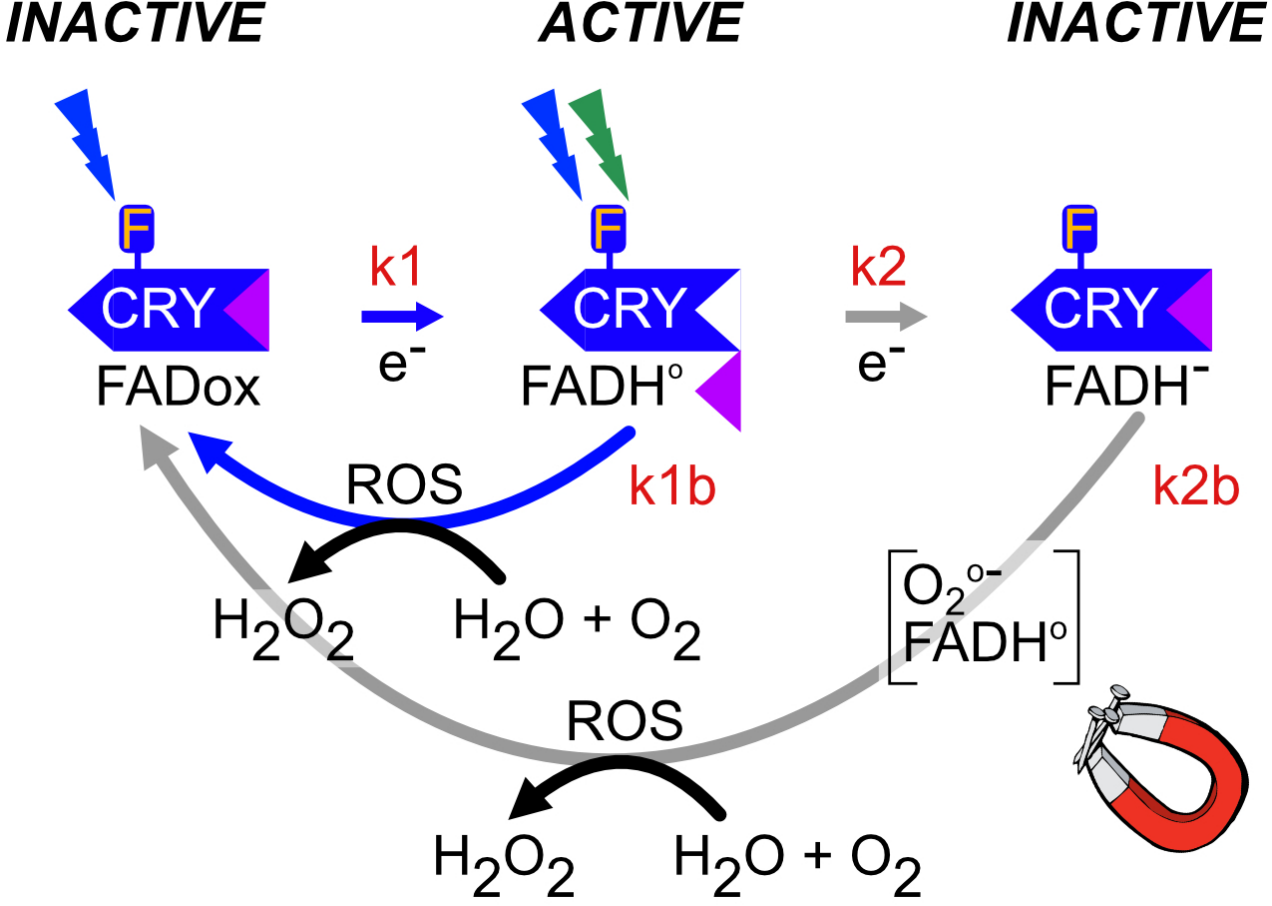
Plant Cry1,2 photocycle. In the dark, flavin is in the oxidized redox state and the protein adopts a biologically inactive conformation (*INACTIVE*). Upon illumination (blue or UV light), the anionic flavin radical is formed (FAD•-),which is then protonated to a relatively stable neutral radical redox state (FADH•), with a half-life of over a minute, which is shown in the figure. These redox state transformations trigger a structural change in the protein in which the C-terminal domain flips out from the flavin binding pocket and becomes accessible to biological sigaling partners (*ACTIVE*). This represents the biologically active (‘lit’) state. Further illumination of the flavin radical (which can absorb either UV, blue or green light) results in formation of the excited radical state (FADH*) followed by reduction to the fully reduced (FADH^-^) redox state shown in the figure. This state is biologically inactive (*INACTIVE*). Both radical (FADH•) and reduced (FADH^-^) flavin undergo slow reoxidation to the resting (FADox) redox state. Biological activity is defined by the equilibrium concentration of FADH•, which is determined from the sum of both light-dependent (k1 and k2) and light-independent (k1b and k2b) rate constants (Procopio et al., 2016). The magnet symbol indicates reactions potentially subject to modulation by magnetic field exposure (flavin reoxidation – Section 5.1).

Cumulatively, all of these changes have led to the evolution of a light-inducible, dark - reversible photoreduction pathway which is at the basis of Cry photoreceptor function. This property of reversible response to light is known as a photocycle (**Figure 4**).

**Figure 5:**
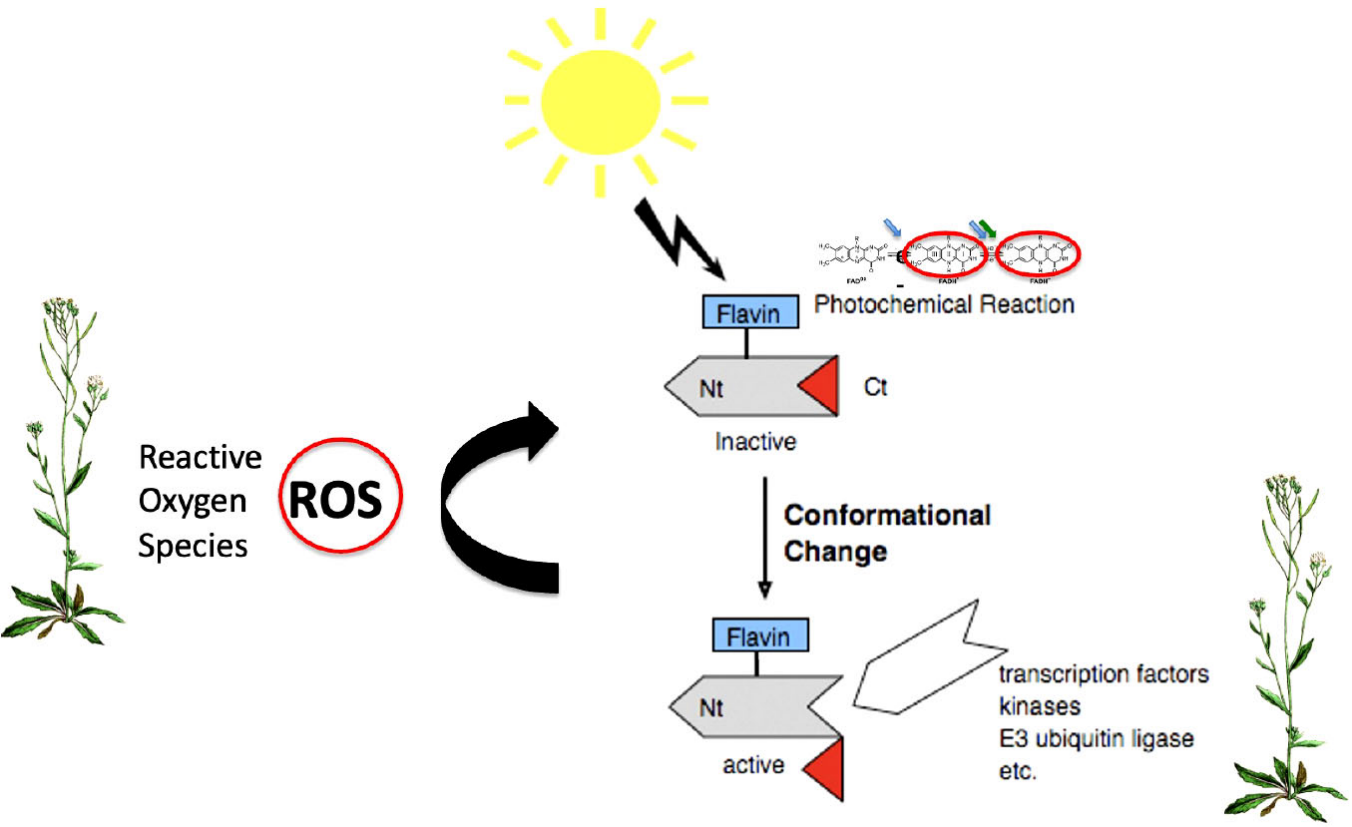
Biological signaling mechanisms of Cry. Light is absorbed through the oxidized flavin chromorphore (FADox) inducing the formation of the radical (FADH•) active ‘lit’ state. Biological activation ensues through conformational change within the Cry protein, leading to unfolding of the C-terminal domain and subsequent access to partner proteins and signaling intermediates (Ponnu and Hoecker, 2022). A second signaling mechanism results from the flavin reoxidation reaction (FADH• to FADox), which occurs in the absence of light and generates ROS (Reactive Oxygen Species) (curved arrow). These in turn can induce ROS-regulated signaling pathways even independently of conformational changes in Crys (El-Esawi et al., 2017).

The plant cryptochrome photocycle has been derived from a variety of physiological and spectrochemical approaches, including the direct detection of flavin redox states *in vivo* using whole-cell EPR and Infrared spectroscopic methods (summarized in Chaves et al., 2011; Ahmad, 2016; Procopio et al., 2016; Goett-Zinc and Kottke, 2021). Briefly, the photocycle is as follows: cryptochrome occurs in the dark with flavin in the oxidized redox state (see **Figure 4**, INACTIVE at top left). In this dark-adapted state, the C-terminal domain is folded within the flavin binding cavity and Cry is biologically inactive. Absorption of blue or UV light by FADox induces flavin photoreduction to the neutral radical state (FADH•) at a rate (k1) that is proportional to the light intensity (Procopio et al., 2016). Photochemically, flavin reduction occurs via intraprotein electron transfer through a chain of conserved Trp and Tyr residues by the process of ‘photoactivation’ as in photolyases (see 2.2). The redox state transition induces a conformational change at the C-terminal domain (Goett-Zink et al., 2021) and renders the protein biologically active (**Figures 4**, **5**). Further illumination induces the formation of the fully reduced redox form (FADH-) (rate constant k2), which is short-lived and biologically inactive in plants.

To restore the resting state, reduced flavin (FADH• and FADH^-^) reacts with molecular oxygen (k2b, k1b) to restore oxidized FADox (*INACTIVE*). This reoxidation reaction occurs independently of light and is accompanied by the formation of Reactive Oxygen Species (ROS) and H_2_O_2_ byproducts. The Cry photocycle can therefore be considered as a dynamic equilibrium between distinct flavin redox states whose relative concentration is governed both by the light intensity (k1 and k2) and by the dark reversion rate (k1b and k2b) (Procopio et al., 2016).

#### 3.2.1 The dark reversion reaction: mechanism of flavin reoxidation

As stated above, one of the unique features of Crys as distinct from photolyases is that they undergo efficient flavin reoxidation *in vivo*. Oxidation of reduced free flavin in solution (FAD(H)•, FADH^-^) to the oxidized state (FADox) is a well-understood reaction that can result in the formation of multiple ROS (reactive oxygen species) intermediates including superoxide (0_2_^-^°), peroxide derivatives and H_2_0_2_ (Romero et al., 2018). Whereas the kinetics for re-oxidation of free FAD flavin are relatively slow (250 M^-1^s^-1^), the oxygen reactivity of protein-bound flavins may be literally orders of magnitude greater (e.g. 2 × 10^6^ M^-1^s^-1^ for glucose oxidase, or 4 × 10^4^ for D-amino acid oxidase) (Massey, 2002). The basis for these astounding differences in efficiency is far from established. However, properties mediated by the protein component such as metal ion binding, stabilization of radical intermediate states, organic ligand binding, oxygen diffusion rates, hydrophobicity, and the charge within the flavin pocket can all have critical effects on reoxidation rates in different flavoenzymes (Massey, 2002).

In the case of plant cryptochromes, protein-bound flavin is reoxidised from the FADH^-^ and/or FADH• radical state by a relatively slow process (k = 50 M^-1^s^-1^) involving the formation of superoxide, flavin hydroperoxide and flavin anionic radical reaction intermediates as well as hydrogen peroxide (H_2_0_2_) as an end product (Müller and Ahmad, 2011; Van Wilderen et al., 2015). Importantly, this relatively slow reoxidation rate (half-life on the order of several minutes) has also been observed to occur *in vivo* using whole-cell EPR spectra of insect cells overexpressing plant crys (Herbel et al., 2013), confirming its biological relevance.

#### 3.2.2 The cryptochrome photocycle is responsive to multiple environmental signals

Light: the photocycle explains how Cry is an effective, reversible light sensor, as biological activity is proportional to the equilibrium concentration of activated (FADH•) redox form, which the light intensity controls through flavin reduction reaction rate constants k1 and k2 (**Figure 4**) (Procopio et al., 2016). Most studies on biological light sensing activity of plant cryptochromes have therefore focused on light-driven forward reaction intermediates either in isolated proteins or *in vivo*.

Other signals: However, as mentioned above, an additional feature of the photocycle is a relatively slow dark reversion rate (k1b, k2b) of several minutes, which is orders of magnitude slower than the light activation step (milliseconds). Since the dark reversion rate (k1b) governs the lifetime of the active form (FADH•), reoxidation rate has a disproportionate effect on biological activity of Crys. This is how external forces that affect reoxidation rates will modulate Cry biological activity, even independently of light. Such tuning forces can include temperature (Pooam et al., 2021); ligand binding (Eckel et al., 2018); localized changes in the midpoint potential of the cellular environment (Romero et al., 2018) or, most intriguingly effects of applied magnetic fields (see 5.1 below).

ROS Signaling: A second potential signaling feature conferred by the reoxidation step is that it generates Reactive Oxygen Species (ROS) as a byproduct of illumination. ROS, and in particular hydrogen peroxide (H_2_0_2_) and superoxide radicals, are versatile signaling intermediates that promote resistance to biotic or abiotic stress, regulation of senescence (ageing), repair of oxidative damage to DNA and proteins, and many other responses. ROS dependent signaling mechanisms are highly conserved, and can include both enzymes and transcription factors that are directly regulated by ROS (e.g. FOXO in C. elegans and humans). They may also stimulate enzymatic activity and ROS scavenging pathways that decreases cellular ROS concentration (e.g. catalase) (Storz, 2011; Schieber and Chandel, 2014). In the case of plant cryptochromes, ROS is formed directly in the nucleus as a byproduct of illumination of both Cry1 and Cry2 (Consentino et al., 2015; Jourdan et al., 2015) and this mechanism has been implicated in the modulation of genes for pathogen defense, and resistance to biotic and abiotic stress (El-Esawi et al., 2017).

In sum, the cryptochrome photocycle confers an exquisitely tunable, versatile biological signaling mechanism that is responsive not only to light wavelength and intensity, but also to extraneous factors that can modulate redox reaction rates. Thus, Crys respond to factors including temperature, pH, cellular metabolites, and even exposure to electromagnetic fields (see section 5). In plant Crys, the primary signaling mechanism is well established to arise from conformational change triggered by FADox to FADH• redox state transition. However, there is also a secondary signaling role for ROS synthesized in the course of the Cry photocycle, which may have important roles in response to stress.

## 4 Biological relevance

The real challenge in assigning any photocycle deduced from *in vitro* photochemical properties is to demonstrate the actual validity of the proposed mechanism *in vivo*. Evidence in support of the biological validity of the plant Cry photocycle has been obtained from *in vivo* spectroscopic, genetic, biochemical, and physiological studies in plants, yeast, and other experimental systems (Chaves et al., 2011; Ahmad, 2016; Hammad et al., 2020).

Here we review the evidence in support of the radical (FADH•) redox state of Crys as the biologically active state, and that any factors that alter the concentration of the FADH• form (such as light) will correspondingly modify Cry biological activity. In addition, we explore the connection between Cry redox state interconversion and the physiological changes that occur in plants.

### 4.1 Evidence that the flavin radical is the ‘lit’ biologically active state *in vivo*

An action spectrum of the physiological response of Cry1 to different wavelengths of light *in vivo* showed the characteristics of oxidized flavin (peak at 450, shoulders near 420 and 480nm, and no significant response above 500nm - green light) (Ahmad et al., 2002). This is consistent with all the known Cry functions in plants, which are activated only under blue/UVA light (Wang and Lin, 2020; Ponnu and Hoecker, 2022).

If the FADH• is the light-activated redox state, any illumination regimen that depletes FADH• should also reduce biological activity. This prediction was validated by co-illumination experiments using both blue and green light together. Whereas blue light drives the reaction FADox to FADH• to accumulate the biologically active signaling state, green light is only absorbed by the radical redox state and thereby drives the reaction: FADH• to FADH^-.^ Therefore co-illumination of green and blue light should deplete FADH• relative to blue light illumination alone. This was indeed observed for both Cry1 and Cry2 (Banerjee et al., 2007; Bouly et al., 2007; see also discussion in Ahmad, 2016). This is indirect evidence that flavin photoreduction to the FADH• redox state activates plant Cry.

Direct evidence for the occurrence of flavin photoreduction *in vivo* involves work by Robert Bittl and Erik Schleicher at FU Berlin. The plant Cry1 and Cry2 photoreceptors were overexpressed in insect cell cultures to achieve a high concentration of receptor protein. Flavin radical formation was subsequently visualized in living cells by whole-cell EPR spectroscopy. This method provided direct evidence of flavin reduction in response to illumination and, using ENDOR analysis, demonstrated the formation of the neutral radical (FADH*) redox state (Banerjee et al., 2007; Bouly et al., 2007; Herbel et al., 2013). Other examples of whole-cell EPR spectroscopy have followed the redox state transition of the Drosophila Cry, which likewise correlates with *in vivo* physiological data (Ahmad, 2016).

Combining physiological methods with *in vivo* spectroscopy has furthermore determined the lifetime of the signaling state of plant Crys *in vivo* (Herbel et al., 2013). This was done by directly measuring the concentration of the signaling state FADH• in living cell cultures over time using EPR spectroscopy. At the same time, the half-life of the biological signaling state *in vivo* was determined by measuring its ‘escape time’, or time after which the biological response was no longer reversible by green light. In this way, the lifetime of the signaling state as measured by biological activity *in vivo* (escape time) was shown to match the half-life of cryptochrome-bound FADH•, consistent with the deduced photocycle.

An additional approach to define the photocycle has been kinetic modeling of the Cry1 and Cry2 redox interconversion rates (**Figure 4**; k1, k2, k1b and k2b), and to compare these rates deduced from *in vitro* studies with purified proteins to those deduced from physiological responses to light in plants. Calculation of the relative quantum yields of the *in vitro* photoreactions showed that flavin photoreduction efficiency and FADH• radical formation was roughly four-fold greater for Cry2 than for Cry1. This calculated greater light sensitivity for Cry2 flavin reduction deduced from ‘*in vitro*’ experiments indeed closely matched the greater light sensitivity of Cry2 *in vivo* (Procopio et al., 2016).

### 4.2 Genetic evidence: mutants in the Trp triad pathway

The first step in the Cry photocycle is flavin photoreduction from the oxidized (FADox) to the biologically active radical (FADH•) redox state. Studies exploring the Cry photoreduction mechanism *in vitro* have established that the Trp triad pathway is used in plant Crys similarly to that in photolyases (see Section 2.2). Mutation in any of the three conserved Trp residues mediating electron transfer from the protein surface to the flavin effectively abolishes the plant cry ability to undergo flavin photoreduction *in vitro* (Zeugner et al., 2005; Engelhard et al., 2014).

Thus, a prediction of the Cry photocycle is that Trp triad mutants of Cry should lose light sensitivity, as they do not form the FADH• radical state.

This prediction was addressed experimentally using Cry1 Trp triad mutants expressed in transgenic plants. The isolated mutant proteins failed to reduce upon illumination *in vitro*, and did not accumulate the active FADH• radical state. When expressed *in vivo*, the photoreduction-deficient Cry1 mutant constructs showed significantly reduced biological activity in mediating plant growth responses such as anthocyanin accumulation and hypocotyl growth inhibition in responses to blue light (Zeugner et al., 2005). Thus, reduced biological activity correlated with reduced FADH• radical state as predicted. Further indication of the effect of the Trp triad mutants on biological function of Crys came from transient grating experiments of M. Terazima. The diffusion coefficient was determined for isolated plant Cry1, before and after illumination. A significant increase in protein volume was observed upon illumination of wild type Cry as measured by its diffusion rate (Kondoh et al., 2011), which are now known results from the light-dependent oligomerization required for biological signaling (Ponnu and Hoecker, 2022). By contrast, Cry1 proteins with mutations in the Trp triad electron transfer pathway showed no such light-dependent increase in density, indicating an absence of conformational change related to signaling (Ponnu and Hoecker, 2022). Taken together, these results using two different experimental approaches from independent labs, both showed correlation of flavin reduction to the radical (FADH•) in order to reach a biologically relevant signaling state.

There has been subsequent confusion as to the effect of the Trp triad in Cry activation resulting from conflicting (to Zeugner et al., 2005) reports that Trp triad mutants of Cry1 and Cry2 were functional as blue light receptors in plants (Li et al., 2011; Gao et al., 2015). These apparent contradictions are however simply resolved by closer inspection of the actual data. In the first paper (Li et al., 2011) the Cry2 Trp triad mutants assigned as being blue light responsive, were in fact also biologically active in red light and darkness, thereby constitutively active and not light responsive at all. We now know from recent CryoEM studies that these mutants in fact had irreversible conformational changes rendering them constitutively active, and unresponsive to light (Hao et al., 2022). Similarly, Trp triad mutants of Cry1 presented as being blue light responsive, only occurred at blue light intensities far above saturation (Gao et al., 2015). In the supplement of this same paper, a proper dose response curve showed almost 95% reduction in blue light sensitivity of Trp triad mutants as compared to the relevant wild type overexpressing controls (see analysis presented in Hammad et al., 2020 for details). Even the residual biological activity of Trp triad mutants was shown to result from residual *in vivo* (FADH•) formed through alternate (to the Trp triad) electron transfer pathways *in vivo* (Engelhard et al., 2014).

In sum, light responsivity correlates with *in vivo* formation of FADH• and is consistent with analysis of the plant Trp triad mutants. It is also clear that the Trp triad plays a structural role in plant Crys apart from its role in electron transfer, particularly in the case of Cry2.

### 4.3 The lifetime of the Cry radical state is correlated with Cry biological activity

Another prediction of the Cry photocycle is that mutations that alter the lifetime of the reduced Cry redox states should affect biological function. Indeed, recent reports of mutations affecting the lifetime of FADH• redox state subsequent to illumination in yeast further support its biological signaling role. A two-hybrid genetic screen was performed using the interaction of Cry2 with its relevant plant signaling partner CIB1 as a direct assay for biological activity (Taslimi et al., 2016). In this screen, performed under blue light pulse conditions, mutants were obtained with enhanced biological activity in response to short intermittent pulse conditions. These mutants mapped to the flavin pocket, and were shown to slow down the rate of flavin re-oxidation from the neutral semiquinone to the oxidized FAD redox state in a related algal CRY. The resulting stabilization of the flavin radical, which would prolong its lifetime under pulsed light conditions, explains the corresponding increase in biological activity in yeast.

Consistent with these findings, the effect of mutations that disrupt the ATP binding site in the plant Cry flavin pocket showed diminished Cry biological activity in transgenic plants *in vivo* (Eckel et al., 2018). The ATP binding site was targeted for mutation as ATP binding is known to significantly prolong the lifetime of the Cry radical (FADH•) redox state both *in vitro* and *in vivo* (Kottke et al, 2021). As predicted, there was diminished biological activity in mutants lacking the ATP binding site consistent with a reduced ‘lit’ state concentration (Eckel et al., 2018). In further support of these experiments, additional mutants slowing the reoxidation rate of plant Cry2 *in vitro* resulted in enhanced biological activity in transgenic plants (Araguirang et al., 2020). These included the L404F substitution mutation in the plant Cry2 which enabled the purified protein to be maintained in a stable radical redox state in the dark *in vitro*. *In vivo*, this mutation conferred constitutive biological activity in the dark as well as hypersensitivity to light, consistent with FADH• accumulation. Importantly, structural studies confirmed that this particular mutation triggered no misfolding artifacts in the protein, unlike the case with the Trp triad mutants – see above and (Li et al., 2011). Therefore, the increased biological activity can be ascribed to the enhanced stability of the flavin radical in this *in vivo* system.

### 4.4 Absence of alternative light sensing mechanisms

One alternative hypothesis to a cryptochrome redox photocycle has been proposed to date, namely that the anionic flavin radical (FAD**•-)** represents the dark-adapted ‘resting state’ of plant Crys, whose illumination triggers a photocycle involving reversible electron transfer from flavin to ATP (see eg. Liu et al., 2010). This suggestion is possibly inspired by the photolyase DNA repair mechanisms, and the fact that the anionic radical redox state is detected in certain mutants of E.coli photolyase (eg N378D) (Müller et al., 2016) and in the insect type I Cry (see eg. Song et al., 2007). However, this mechanism is not consistent with any of the available evidence for plant Cry. The anionic radical formed in the course of flavin reduction exists only transiently, as it is rapidly and efficiently protonated by the conserved proximal D residue in the flavin pocket (D396 or D393) to form the stable neutral radical FADH• (Burney et al., 2012). There is furthermore no evidence for existence of a stable anionic radical (or indeed for any plant Cry radical flavin state) in dark-adapted cells *in vivo*, either indirectly or by direct whole-cell spectroscopic means (Bouly et al., 2007; Herbel et al., 2013; Goett-Zink et al., 2021). Finally, ATP binding as required by this hypothesis is not essential for Cry biological activity *in vivo* (Eckel et al., 2018).

Nonetheless, it cannot be excluded that additional reactions occurring in the framework of the plant Cry redox photocycle may tune photosensory efficiency and/or respond to different environmental cues. Possible contribution of additional photoreactions to the signaling mechanism of plant Crys therefore awaits experimental evidence.

### 4.5 How the photocycle triggers structural change linked to biological function

A key ‘missing link’ in the mechanism for Cry light activation are the intermediate steps between flavin photoreduction and the conformational change that occurs in plant Crys that form the basis of the biologically active ‘lit’ state. The problem is challenging as flavin does not itself undergo large conformational changes upon reduction.

Recent clues concerning these intermediate steps have come from experiments on the *Drosophila* Cry showing that release of the C-terminal domain from its docking position within the flavin binding pocket can be triggered by photoreduction of flavin. The ensuing conformational change in the protein was initiated through changes in charge and protonation state of a conserved His378 residue in close proximity to the flavin (Lin et al., 2018), which, significantly, did not induce large structural changes within the flavin pocket. Further indirect support for the possibility that subtle electrostatic changes within the flavin pocket could initiate conformational change was obtained using high-resolution serial crystallography of flavin photoreduction in photolyases (Maestre-Reyna et al., 2022). These showed that the formation of the anionic radical induces a strong twist in the isoalloxazine ring which triggers ensuing electrostatic perturbations in the flavin pocket, leading to structural changes at the protein surface.

#### 4.5.1 Light-induced structural changes in plant-like Crys as detected by FTIR spectroscopy

In the case of plant Crys, time-resolved experiments using IR spectroscopy have been used to probe structural changes resulting from flavin photoreduction in the plant-like Cry1 from *Chlamydomonas rheinhardtii*. They show conformational change is initiated within the N-terminal photolyase-like domain of Cry, likely due to electrostatic charge fluctuations in the flavin pocket triggered by photoreduction (Langenbacher et al., 2009). These structural changes occur within microseconds of illumination, in the β sheet region proximal to the flavin. This time scale is consistent with formation of the anionic radical (FAD•-). (Thöing et al., 2015; Goett-Zinc and Kottke, 2021), and the structural changes persist over several minutes subsequent to illumination, consistent with the lifetime of the signaling state (FADH•).

As a consequence, the C terminal domain (CCT), which is folded against this β sheet region in the dark-adapted Cry state, subsequently unfolds and is exposed at the protein surface consistent with its known signaling role (Goett-Zink et al., 2021). In this way, signal propagates from formation of FAD•- to the β-sheet bound to Cry CCT. The resulting reorganization induces the dissociation of the CCT from the PHR – like N-terminal domain, and the activated conformation stabilized in the presence of FADH• (Goett-Zink et al., 2021). All these changes fit with the proposed downstream signaling mechanism of higher plant Crys, which involves unfolding and exposure of the C-terminal domain (CCT) and subsequent oligomerization followed by interaction with cellular signaling intermediates (Wang and Lin, 2020).

#### 4.5.2 Structural changes in Cry as detected by CryoEM

Direct structural information on the biologically active form (‘lit state’) of plant Crys has been obtained from CryoEM studies on the N-terminal domains of light-activated plant Cry2 (Ma et al., 2020; Palayam et al., 2021). These studies have identified amino acid residues mediating tetramerization of Cry2, as well as residues that interact with the Cry inhibitor BIC2 (Kruusyee et al., 2022), as a result of protein restructuring subsequent to illumination. Flavin reduction to the neutral radical FADH• redox form was thought to cause the weakening of the hydrogen-bonding interactions between amino acids in close proximity to the flavin. The subsequent loosening and enlarging of the FAD-binding cavity was proposed to trigger modifications in the α/β-domain, the connector region, and the α-domain promoting the formation of CRY2 oligomers and subsequent biological activation. These results were in agreement with prior time-resolved FTIR studies by Goett-Zinc et al., 2021. Similar modifications were observed in CryoEM structures obtained from the dark-adapted form of *Arabidopsis* Cry2 Trp triad mutant (W374A) and its interaction with the partner protein CIB1 (Liu et al., 2020; Hao et al., 2022). This mutant showed light-independent biological activity (Li et al., 2011), caused by structural perturbations that mimic those of the wild type light-activated form. Structures for the rice (Zea Mays) oligomerized Cry1 protein (Shao et al., 2020) also showed expansion of the flavin cavity upon light activation. Thus, evidence from independent groups shows structural change in the flavin cavity, consistent with past work on algal Crys and photolyases (see eg. Maestre-Reyna et al., 2022).

The challenge remains to detect the intermediate steps that occur between light absorption by flavin in the flavin pocket and the ensuing large structural changes in the α/β-domain, the connector region, and the α-domain. These intermediate steps showing initiation and progression of conformational change await higher resolution and time-resolved experimental methods such as serial X-Ray crystallography as performed with photolyases (Maestre-Reyna et al., 2022), or by a combination of single-molecule Förster resonance energy transfer, structural predictions, and solution X-ray scattering methods as recently reported for the light-triggered conformational change of the animal type CraCry from algae (Li et al., 2022). One major hurdle has been that, although the initial structural change predicted for the plant Cry is the dissociation of the C-terminal domain from the flavin pocket (Wang and Lin, 2020), it has not been addressed by CryoEM. The C-terminal tail of Crys adopts a random coil state (Parico and Partch, 2020), and there is as yet no crystal structure of the full-length plant Cry protein. Since the C-terminal domains of CRYs are unstructured, they may adopt different conformations according to their interaction partners. Co-crystallization studies with different interacting proteins, perhaps via cryo-EM, may be a way to study their structural diversity.

## 5 Special effects related to the Cry photocycle

The Cry photocycle provides an exquisitely tunable, versatile signaling mechanism based on redox chemistry that is sensitive to multiple inputs in addition to light. It is moreover relevant to organisms throughout the biological Kingdom as many fundamental features of Cry photochemistry appear to be conserved. Although a thorough analysis of potential applications and consequences of the cryptochrome photocycle is beyond the scope of this review, a few selected examples of how understanding the photocycle informs scientific advances and novel applications in plants are presented below.

### 5.1 The cryptochrome photocycle and response to applied magnetic fields

The idea that cryptochromes could function as magnetoreceptors came initially from studies with migratory birds, which use the earth’s magnetic field as a compass in seasonal migration. Birds were found to orient preferentially in short-wavelength light (below 600nm) and moreover used an inclination compass which could detect the angle of the earth’s magnetic field but was not responsive to the north-south direction. Quantum physical principles suggested that such a ‘chemical magnetoreceptor’ must undergo a reaction mechanism that generates radical pairs (Ritz and Schulten et al., 2000; Hore and Mouritsen, 2016). As a consequence, cryptochrome, which is localized in the bird’s retina, was suggested as a possible avian magnetoreceptor. A series of famous behavioral and histochemical experiments followed in which the light-sensitivity and spin chemical properties of the avian magnetic compass were related to the light-sensitivity and function of cryptochrome 1a in the avian retina (Wiltschko et al., 2021).

There have also been long-standing observations of plant responses to magnetic fields dating back over a hundred years. Concrete evidence implicating plant cryptochromes in sensitivity to magnetic fields was first documented for plant growth experiments, showing that the effect of a static magnetic field of 500 μT (10-fold the earth’s magnetic field strength) was to decrease seedling growth in blue light (Ahmad et al., 2007). This response was observed in blue/UV light but not in red light, and moreover failed to occur in cryptochrome mutant seedlings consistent with a role for cryptochromes in this response. Although generating initial controversy (Harris et al., 2009), independent labs subsequently upheld a role for both Cry1 and Cry2 in modulating blue-light dependent plant growth responses to static magnetic fields (see e.g. Xu et al., 2012; Xu et al., 2014; Agliassa et al., 2018). Evidence for cryptochrome mediated physiological responses to applied magnetic fields in plant, Drosophila, as well as in mammalian cell systems has been recently summarized in this Review Series (Kyriacou and Rosato, 2022; Zhang and Malkemper, 2023; Thoradit et al., 2023) although areas of controversy exists in all of these cases (see eg. Bassetto et al., 2023).

#### 5.1.1 The radical pair mechanism and Cry magnetosensing

Magnetic fields of the order of the geomagnetic field (30 – 50 μT) are far too weak to initiate chemical reactions. However, a theoretical explanation for how biochemical reactions could nonetheless be manipulated by magnetic fields plays upon the fact that these weak forces can interact with electron spins of radicals in an oriented manner. This is known as the Radical Pair mechanism of biological magnetosensing (Hore and Mouritsen, 2016), and predicts modest (less than 10%) changes in rate constants of susceptible reactions. It can be simply explained as follows: Free radicals are formed in the course of many biochemical reactions as high-energy intermediate states. The energy comes from making and breaking chemical bonds, not from the magnetic field. However, the magnetic fields can intervene, in certain favorable configurations, by changing the electron spins of the high-energy excited state intermediates. In this way, the efficiency of the biochemical or photochemical reaction and the rate of product formation is altered. In biological terms, the result is an apparent change in the rate constant and therefore light sensitivity of a photoreceptor. For example, in the case of cryptochromes, if the magnetic field were to induce a change in any one of the rate constants k1, k2, k1b or k2b (**Figure 4**), then this would be perceived by the organism as a change in light intensity even though the actual number of photons reaching the receptor is exactly the same. The change in efficiency of forming the FADH• signaling state would result in an altered perceived light signal.

The challenge in proving such a mechanism is, first, to demonstrate that it indeed occurs and, second, identifying the magnetically sensitive radical pairs leading to changes in biological activity.

The validity of the Radical Pair mechanism has been directly demonstrated in model compounds and even shown to occur in photoreactions of isolated cryptochromes and photolyases *in vitro* (Hore and Mouritsen, 2016). A further fruitful line of evidence for the Radical Pair mechanism has been the effects of radiofrequency (RF) signals in the MHz range, which were found to disrupt magnetic sensitivity in a number of organisms, consistent with a radical pair mechanism as found in birds (Hore and Mouritsen, 2016; Wiltschko et al., 2021). Such effects are also reported in plants, where exposure to RF was found to disrupt magnetic sensitivity in plant growth phenotypes and to mimic the effect of removing the earth’s magnetic field entirely (Albaqami et al., 2020). All of these data are consistent with a radical pair mechanism, but do not resolve the question of whether cryptochrome really function as a magnetoreceptor in a biological system? And if so what radical pairs are involved?

#### 5.1.2 What is the magnetically sensitive step?

There are at least two known steps within the Cry photocycle that can form radical pair intermediates. The first occurs during the initial reaction of light-driven electron transfer to the flavin, whereby Trp/FADH· and Tyr/ FADH· radical pairs are transiently generated as intermediates. These radical pairs can be triggered solely by absorption of short-wavelength blue or UV/A light with a sharp cutoff at 500nm, as oxidized flavin does not absorb in a spectral range above 500nm (Maeda et al., 2012). As we have seen, the primary photoreduction reaction generates the radical redox state and the biologically active form of the receptor (**Figure 4**, rate constant k1). A further critical characteristic of this reaction step is that it can only occur during illumination; the radical pairs are unstable with half lives in the range of milliseconds (Maeda et al., 2012). Therefore, magnetic field effects could only occur during illumination if this mechanism is involved.

This forward photoreduction reaction was initially favored as a possible target for applied magnetic fields as it showed magnetic field sensitivity *in vitro*. The plant Cry1, for instance, responds to magnetic fields in the mTesla range *in vitro* by altering the quantum yield of radical (FADH•) formation under illumination (k1 rate constant, **Figure 4**) (Maeda et al., 2012). Subsequently, similar results were shown for other Crys and photolyases, including for one of the crys found in the bird retina (avian Cry4) (Xu et al., 2021). These experiments were supported by numerous theoretical papers ‘proving’ how variations of Trp/Flavin radical pairs are a suitable target for modulation by magnetic fields (summarized in Hore and Mouritsen, 2016).

However, this reaction mechanism is in contradiction to the available biological evidence. There are literally dozens of behavioral studies on avian magnetosensing, including from independent labs, that have shown that birds can orient under wavelengths (567nm) where the Trp/FADH• radical pair does not exist. Oxidised flavin does not absorb above 500nm, so cannot be activated by 567nm green light of these avian orientation studies. Further experiments with birds were performed under pulsed light conditions. In these experiments, magnetic field exposure was controlled separately from the light pulses. It was demonstrated that birds could orient even if the magnetic fields was given during dark intervals between light pulses – which is a physical impossibility if the Trp/Flavin radical pair was involved (summarized in Wiltschko et al., 2021).

Further evidence that the magnetically sensitive step mediated by the cryptochrome photoreceptor occurs in the absence of light has also been obtained in plants, where magnetic field sensitivity occurs during intervals of darkness between or after illumination periods (Xu et al., 2014; Hammad et al., 2020; Pooam et al., 2021). In some of these experiments there are more than 10 second intervals between the end of the light period and the beginning of the magnetic field exposure. Since the Trp/FADH• radical pair has a lifetime of just a few milliseconds, it cannot mediate magnetic field sensitivity of plant Crys. In other words, as long as an initial step occurs that generates the flavin radical (FADH•), the magnetically sensitive step can be de-coupled from illumination.

Further support for these observations came from cryptochrome-dependent magnetic field effects in the *Drosophila* system. These were likewise observed to occur in the absence of the Trp/FADH• radical pair (Kyriacou and Rosato 2022; Bradlaugh et al 2023). In this case, the N-terminal flavin-binding domain of *Drosophila* Cry is not even required for magnetic sensitivity, such that the needed flavin radical is thought to be generated in other flavoproteins that interact with the *Drosophila* Cry C-terminal domain. This provides a new paradigm for Cry-dependent magnetosensing consistent with a redox-related radical pair mechanism.

Intriguingly, a lab without prior publications on *Drosophila* has recently reported being unable to replicate these experiments (Bassetto et al., 2023). However, this same lab likewise failed to replicate other behavioral studies they attempted, claiming to discredit all prior findings on *Drosophila* Cry as a magnetoreceptor (which have been published over the last 15 years). As many of these studies had already been replicated by independent, leading *Drosophila* labs (see eg. Yoshii et al., 2009; Fedele et al., 2014; Bae et al., 2016; Kyriacou and Rosato, 2022), a visit to a *Drosophila* lab with a working behavioral assay system by Basetto and colleagues may be the most effective means to resolve their difficulties (see also Section 5.4 below).

#### 5.1.3 Flavin reoxidation and formation of a radical pair

As stated above, the physiological evidence in plants points to a magnetosensing mechanism in which light is required for Cry activation (reaction step k1 in **Figure 4**), but in which the actual magnetically sensitive step occurs during the dark reoxidation reaction subsequent to illumination (Hammad et al., 2020; Pooam et al., 2020; Pooam et al., 2021; Albaqami et al., 2020). Because flavin reoxidation produces ROS, Reactive Oxygen Species intermediates including hydrogen peroxide and superoxide radicals O_2_·- together with FADH• have been suggested as possible radical pairs (Müller and Ahmad, 2011).

At present, the precise nature of the radical pair formed by plant Crys in the course of magnetic field exposure has not yet been determined. As they are formed exclusively in the process of flavin reoxidation they likely involve the flavin radical and some species of reactive oxygen (Müller and Ahmad, 2011). There are theoretical constraints suggesting that superoxide, which is formed during the flavin reoxidation step, is an unlikely candidate to participate in magnetoreception, since the spin relaxation time is too rapid (Hore and Mouritsen, 2016). However, alternative mechanisms, such as involving scavenging radicals or other intermediates have been suggested (Kattnig and Hore, 2017; Rishabh et al., 2022) which await experimental verification.

### 5.2 Magnetosensing mechanisms that may indirectly involve Crys

The evidence shows that plant Crys likely function as direct magnetoreceptors (Pooam et al., 2019; Hammad et al., 2020), as it is possible to visualize effects of magnetic fields directly on receptor phosphorylation and Cry conformational state *in vivo*. These effects occur *in vivo* within minutes of magnetic field exposure and can be most readily resolved under pulsed light conditions.

However, it is becoming increasingly apparent that there are additional mechanisms for plant magnetosensing, which may involve Crys only indirectly, but not as the actual magnetosensors. For instance, plant cryptochrome mutants showed altered gene expression profiles in a magnetic field as compared to wild type plants, even in the complete absence of light (Dhiman and Galland, 2018). This surprising result would implicate plant Cry in magnetic field sensing, but without activating the photocycle. Such indirect mechanisms involving Crys but not the Cry redox photocycle, have also been reported in flies (Kyriakou and Rosato 2022). Some of these may involve mitochondrial redox reactions, that likewise respond to magnetic fields by the radical pair mechanism, and do so independently of light (Usselman et al., 2016; Ikeya and Woodward, 2021).

A further alternate magnetosensing mechanism involving Crys has been the suggestion that ferromagnetite/cryptochrome complexes called MagR may function in plant magnetic field sensing (Xie, 2022). This suggestion is based on the observation that MagR, a conserved iron-sulfur binding protein originally named IscA1, co-purifies with Cry in both animal and plant species. Because ferromagnetite can sense (align with) the polarity of the magnetic field in bacteria and some animals, it was speculated that magnetic field sensitivity of Crys could also be due to bound magnetite as the magnetosensing party (Xie, 2022). However, this MagR mechanism is in contradiction to known Cry-dependent responses to the intensity of the magnetic field but not its direction. Furthermore, RF (radiofrequency fields) have been shown to interfere with Cry-dependent magnetic field perception in plants (Albaqami et al., 2020), which is a diagnostic feature of the Radical Pair mechanism. Such RF interference cannot be mediated through a magnetite-based mechanism. Although it cannot be excluded that plants have some form of magnetite-based magnetic sensitivity mechanism, experimental proof for MagR and magnetite based magnetosensing is currently lacking.

### 5.3 Plant magnetic field sensitivity by mechanisms that do not involve the Crys

Intriguingly, mutant analysis suggests that other plant photoreceptors distinct from cryptochromes may be implicated in magnetic field effects on plant growth and gene expression, even in the absence of light. These include phytochrome and phototropins (Agliassa et al., 2018; Dhiman and Galland, 2018), neither of which can form radical pairs as reaction intermediates, nor are they known to function in the absence of light. Though unlikely to function as direct magnetosensors, gating by a variety of photoreceptors sets up new paradigms for magnetic field sensitivity as well as identifying new roles for plant photoreceptors that occur in the absence of light. The actual magnetosensing mechanism in these cases remains unknown, however.

### 5.4 Resolving areas of controversy

The field of magnetobiology has historically been plagued by controversy and contradictory findings and therefore has had a somewhat dubious past (see eg. Galland and Pazur, 2005). As even this brief analysis shows, there appear to be multiple unrelated magnetic field response mechanisms acting simultaneously in plants, and affecting different biological properties to differing degrees. This complexity is compounded by the fact that magnetic field effects are often quite subtle (predicted primary effects of the Radical Pair mechanism are less than 10% change in reaction yield). These kinds of effects require an exceptional mastery of the experimental system to reliably visualize (eg. bird orientation, *Drosophila* behavioral experiments, plant growth experiments). Finally, artifacts due to poor characterization of experimental conditions are compounded with everyone using completely different readouts and exposure conditions, making comparison among past studies virtually impossible in many cases (Galland and Pazur, 2005).

Today, improving methodology has achieved better controlled conditions and resulted in reliable results and greater reproducibility between different labs, particularly for bird, plant (*Arabidopsis*), and *Drosophila* magnetic field exposure experiments. For example, cryptochrome mediated magnetic field effects on plant seedling growth that are dependent on blue light have been extensively replicated (Ahmad et al., 2007; Xu et al., 2012; Xu et al., 2015; Agliassa et al., 2018; Pooam et al., 2019; Hammad et al., 2020; Albaqami et al., 2020). Current disputes can often be resolved on purely methodological grounds (failure to replicate results due to failure to precisely replicate the experimental conditions under which they were obtained). For example, a reported inability to observe plant blue light-dependent and Cry-dependent magnetic field sensitivity in plants (Dhiman and Galland, 2018; Dhiman and Galland, 2018) can be ascribed to the profoundly different experimental conditions and assays used (as compared to successful experiments presented in Xu et al., 2012; Xu et al., 2015; Agliassa et al., 2018; Albaqami et al., 2020). Thus, strict attention to methodological parameters, possibly including visits to the respective labs should help make methodological issues a thing of the past (and resolve disputes framed by studies such as Bassetto et al., 2023).

A second source of controversy concerns interpretation of findings rather than of the data itself. An ongoing example of such confusion is a continuing stream of publications describing short-lived Trp/flavin radical pairs of Crys as mediators of biological magnetic field sensitivity (for analysis, see Section 5.12 above). This is in contradiction to all of the behavioral evidence: birds are oriented in green light and in darkness, where formation of Trp/flavin radical pairs by Cry is a physical impossibility (see eg. Wiltschko et al., 2021). This impossible mechanism has even recently been proposed as the basis for the avian magnetic compass (Xu et al., 2021), and has been widely disseminated as such in the popular press. Because magnetoreception is a highly interdisciplinary field, such confusion may occur when physicists and chemists do not consider biological data, or vice versa.

However, in order to be valid, a proposed mechanism must first and foremost be consistent with the physiological response. The biological evidence must always take precedence over ever other form of analysis. Truth is a zero-sum game: if something is correct (such as that birds navigate in green light), then a contradictory hypothesis (ie. that Trp/flavin radical pairs of Crys mediate magnetic orientation) must be false, no matter how attractive it may be on theoretical and *in vitro* experimental grounds. To quote the founders and current leaders in the field of bird navigation, Wolfgang and Roswitha Wiltschko: ‘the bird is always right’.

## 6 Emerging technologies involving the cryptochrome photocycle

Cryptochromes impact on practically every aspect of plant growth and development. They can influence seed germination, flowering, plant architecture, fruit metabolic content and resistance to biotic and abiotic stresses (Chaves et al., 2011). Accordingly, cryptochromes have broad potential as targets for adaptive modifications of crop plants (Fantini and Facella, 2020); or through stimulation through electromagnetic fields or other forces to improve crop yield and resistance to biotic and abiotic stress including those related to climate change (Pooam et al., 2020).

Plant cryptochromes have in addition been developed as potent optogenetic tools for regulating gene and protein expression for research and in medicine. For example, Cry2 has been used as a light-induced oligomerization module (see eg. Konermann et al., 2013). These reversible, light-inducible complexes form the basis of light-sensitive tools for gene expression and sequestering heterologous proteins in both mammalian cells and yeast.

Finally, interest in imaging of quantum forces has stimulated the development of new technologies which may eventually permit visualization of magnetic field effects at the single molecule and nanoparticle scale in living tissue (see eg. Winkler et al., 2023; Passian and Siopsis, 2017). This will help resolve the ongoing problem of relating quantum effects to *in vivo* biological responses.

Although a thorough analysis of these topics is beyond the scope of this review, we provide a brief snapshot of two exciting emerging technologies related to applications and visualization of the cryptochrome photocycle; namely optogenetic control of gene expression in yeast for synthetic biology; and development of diamond sensors for eventual visualization of *in vivo* magnetic field sensitivity of cryptochromes in living cells.

### 6.1 Applications for synthetic biology in yeast

Cryptochromes have significant potential applications in synthetic biology, involving the use of light to control gene expression. Since the yeast *Saccharomyces cerevisiae* (baker’s yeast) is commonly used as a chassis for synthetic biology approaches aiming to produce proteins or high-value chemical compounds, here we will review current efforts in the field to develop cryptochrome-based optogenetic tools for yeast.

In yeast cell factories, typically, the genes encoding the heterologous biosynthetic pathway are under inducible control in order to avoid product toxicity or draining of cell resources during the growth phase. Inducible gene expression usually relies on the use of promoters that are activated by the presence of specific chemical compounds (e.g., galactose). This can impose limitations since the inducer is difficult to remove once added while tuning of the promoter activity is frequently difficult to achieve with precision. These challenges can be overcome using light-controlled induction of gene expression, which is readily reversible, dose-dependent, non-toxic, and cost-effective. Yeast is a highly suitable chassis for the construction of optogenetic circuits as it does not contain any native photoreceptors and it can provide endogenous flavins for functional cryptochrome expression.

**Figure 6:**
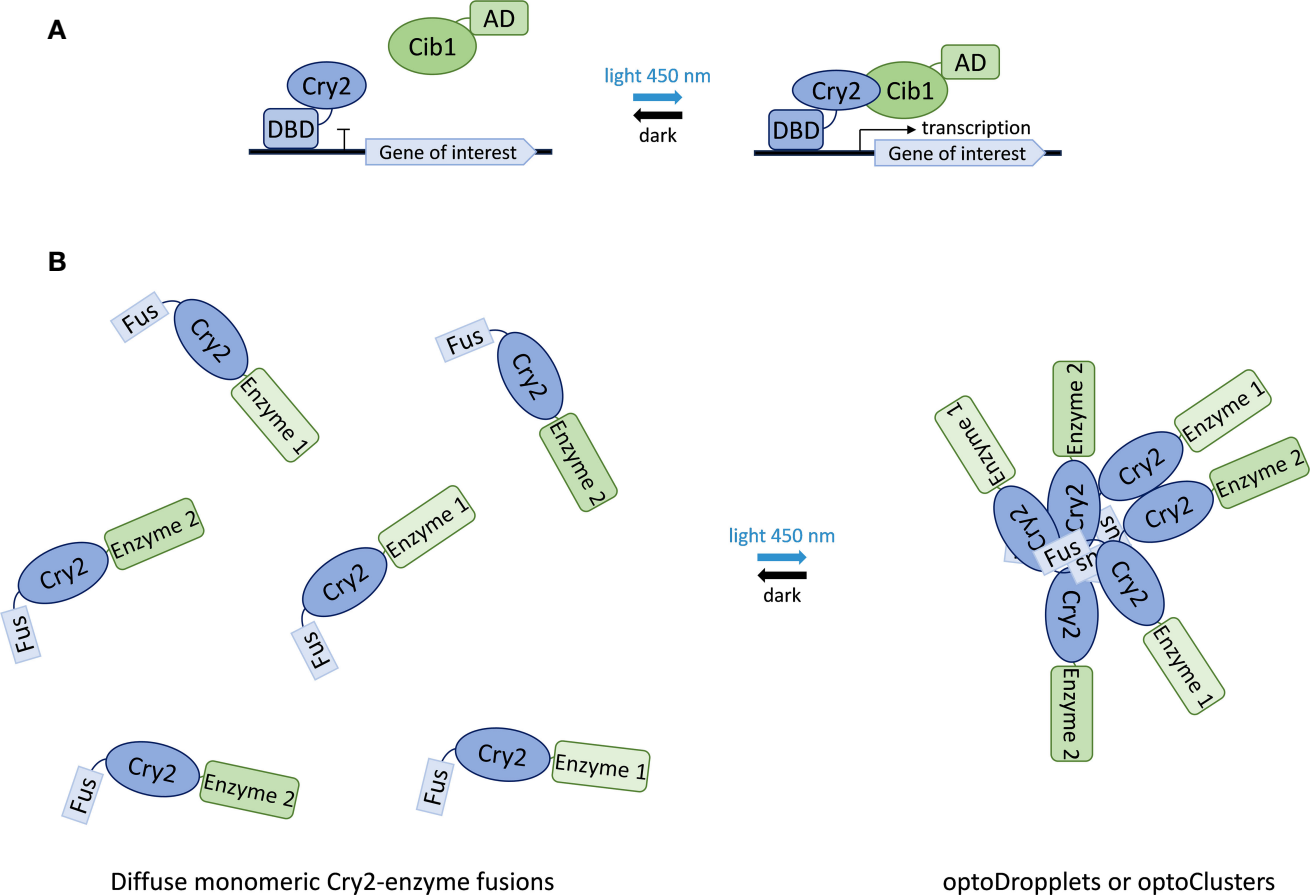
Applications of Cry2 in synthetic biology. **(A)** Light-controlled gene expression switch composed of Cry2 fused to a transcription factor DNA binding domain (DBD) and Cib1 fused to a transcription activation domain (AD). Illumination with blue light causes Cry2-Cib1 association leading to the activation of transcription. Reversion to the dissociated state upon light removal (dark state) stops transcription. **(B)** Formation of optoDropplets and optoClusters based on the association of Fus-protein domain fusions of Cry2 results in the sequestration of attached enzymes upon light illumination.

Thus, fusions between cryptochromes and transcription factor domains have been used to establish synthetic light-inducible transcription factors that control the expression of genes in response to blue light. Most systems take advantage of the *Arabidopsis thaliana* cryptochrome 2 protein (Cry2) and its interacting partner Cib1. By fusing Cry2 with the DNA-binding domain of a transcription factor (DBD) and Cib1 with a transcriptional activation domain (AD), an opto-switch is created that assembles upon blue-light stimulation and drives the expression of the target gene (Liu et al., 2008; Kennedy et al., 2010; Hughes et al., 2012; Melendez et al., 2014; Pathak et al., 2014; Hallett et al., 2016; Taslimi et al., 2016). When blue light stimulation is removed (darkness), the complex dissociates and gene expression ceases (**Figure 6**). This yeast opto-switch has been optimized by the discovery of a photocycle mutant of Cry2 that exhibits reduced interaction with Cib1 in the absence of blue light (Taslimi et al., 2016). Further optimization of this system was achieved by employing a range of different constitutive promoters to express its components, allowing fine-tuning of the sensitivity and responsiveness of the system (An-Adirekkun et al., 2020) (**Figure 6**).

Beyond gene expression, the Cry2 photoreceptor has also been used to control the assembly of enzyme complexes in the context of synthetic compartmentalization and metabolic channeling. This approach takes advantage of the ability of Cry2 to form oligomers upon blue-light stimulation (Yu et al., 2009). This assembly is enhanced by a point mutation in Cry2, giving rise to the variant Cry2olig (Taslimi et al., 2014), and by fusing it to the N-terminal intrinsically disordered region from the protein Fus (Shin et al., 2017). Combination of these two enhancing elements results in two systems with different physical properties. Fusion of the N-terminal region of Fus to wild-type Cry2 forms liquid-like spherical droplets, named optoDroplets, that rapidly exchange monomers in and out of clusters. Fusion of the Fus N-terminal domain to the Cry2olig variant results in rigid clusters that do not exchange subunits with the solution. The latter are called optoClusters (**Figure 6**). Using the deoxyviolacein biosynthesis pathway as a model system, the optoCluster system was shown to enhance product formation two-fold by decreasing the concentration of intermediate metabolites and reducing flux through competing pathways (Zhao et al., 2019). Thus, Cry2-based inducible compartmentalization of enzymes into synthetic organelles is an efficient strategy to optimize engineered metabolic pathways.

In sum, understanding of the cryptochrome photocycle will enable the design and development of novel optogenetic tools impacting on many different synthetic biology applications in yeast. It may even help in the design of novel Cry variants displaying controlled response to external magnetic fields. In combination with suitable lighting protocols, this new technology of ‘magnetogenetics’ could exponentially increase the synthetic biology toolkit.

### 6.2 Novel sensors for measuring Cry activation *in vitro and in vivo*

**Figure 7:**
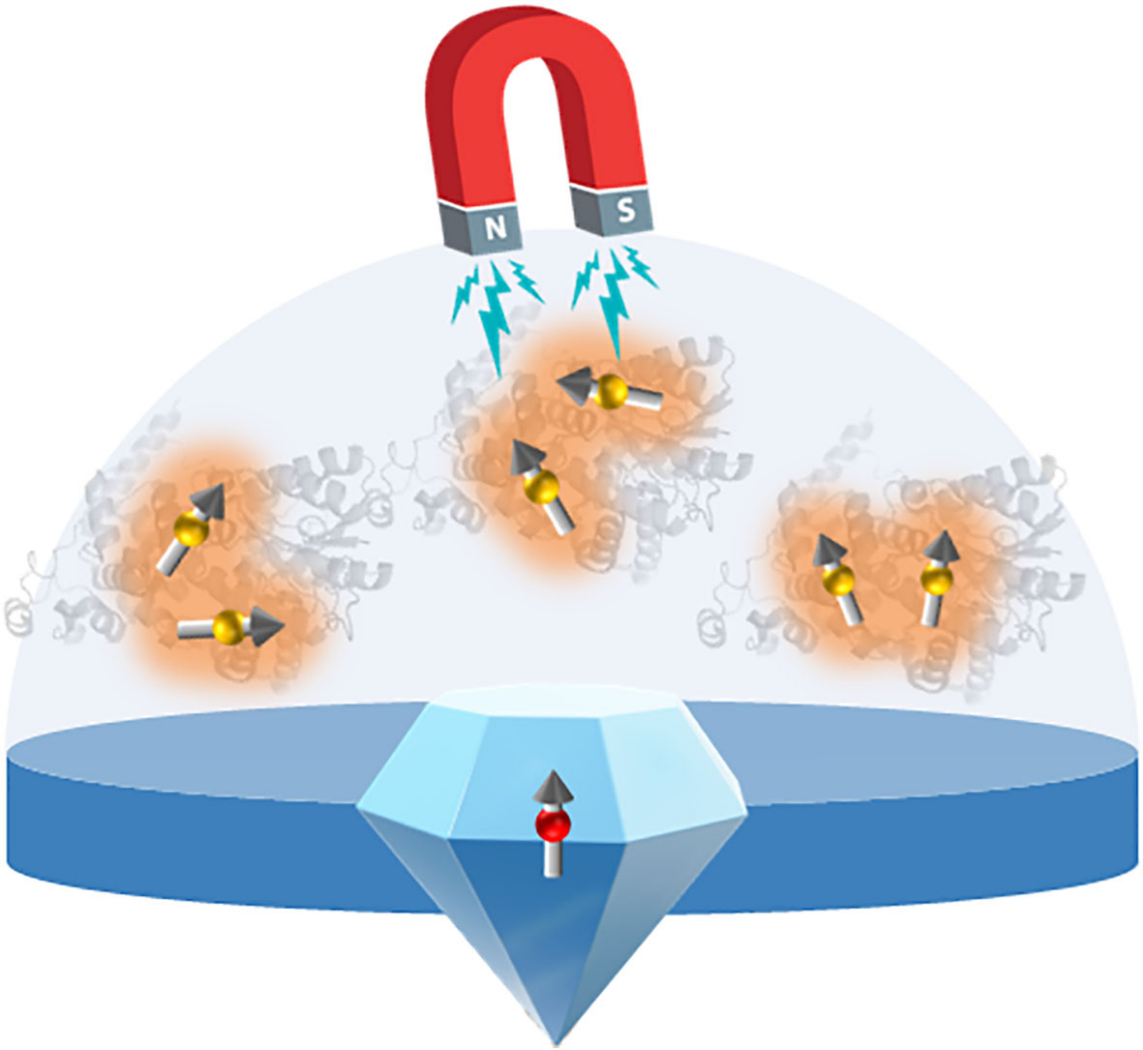
Principle of an NV-center in diamond experiment for *in vitro* sensing of the existence of radical pairs as well as their magnetic field effect. The NV-center in diamond provides the electron spin (arrow with red dot within the diamond-shape) which interacts with the electron-spins of the radical pair molecules (shaded orange regions with arrows with yellow dot). An external magnetic field – here symbolized by a U-shaped magnet - may be applied with varying strength, allowing for the investigation of magnetic field effects.

During the last decade, new bio-sensing schemes based on color centers in diamond crystals have appeared. The most common of these is the nitrogen-vacancy (NV) center which consists of a substitutional nitrogen atom and an adjacent lattice vacancy (Schirhagl et al., 2014; Aslam et al., 2023) (**Figure 7**). As a key feature, the NV center carries a free electron coupled to the optical transition, allowing for both electron spin polarization and readout. By recording electron spin precession, the system thus provides the ability for optical detection of local magnetic field at room temperature. In addition, the diamond system benefits from its high biocompatibility. With mm-sized diamond crystals hosting surface-layers enriched in NV-centers, or nanodiamonds with few or single NV centers, high sensitivity for detection of radicals has been demonstrated (Tetienne et al., 2013; Nie et al., 2021). In contrast to time-resolved electron paramagnetic resonance spectroscopy or optical methods based on absorption or fluorescence detection, such NV-center in diamond-based detection does not necessarily rely on ensemble averaged information of the spin dynamics. This type of sensor should be suitable for the detection of magnetic field effects in relation with cryptochromes, for which numerical investigations suggest a sensitivity allowing for detection of magnetic field effects in even one or few molecules.

## 7 Summary and concluding remarks

Cryptochromes are complex and versatile flavoprotein receptors that have evolved many important signaling functions in both plants and animals. Despite an enormous diversity in origin and in their biological roles, the underlying structure and primary photochemical reactions among cryptochromes are remarkably well conserved. This suggests that detailed understanding of key biological mechanisms in one experimental system may have relevance for all.

We accordingly focus on a critical step in the evolution of plant cryptochromes from their photolyase ancestors, which enabled them to mediate physiological responses to light. This has been the development of a redox cycle in which flavin is reduced by light to a biologically activated ‘lit’ state, followed by spontaneous reoxidation back to a biologically inactive dark-adapted state. This so-called photocycle (**Figures 4**, **5**) provides the basis for plant cryptochrome light sensitivity, and in addition provides a mechanism by which plant cryptochromes can respond to other environmental cues including temperature, applied magnetic fields and even radiofrequency fields.

Furthermore, these Cry signaling mechanisms have been reviewed specifically through the lens of their potential biological effectiveness *in vivo* (**Figure 5**). For example, many photoreactions of plant cryptochrome that have been extensively studied *in vitro* are actually of little or no physiological significance *in vivo*. These include the exhaustively studied flavin photoreduction reactions showing magnetic field sensitivity *in vitro*, but which are not relevant to magnetosensing by Cry *in vivo*. Conversely, certain key aspects of the Cry photocycle that have been largely overlooked during *in vitro* studies, do in fact play a disproportionate role in physiological responses *in vivo*. These include the flavin reoxidation reaction which contributes both to the magnitude of the biological response and to magnetic field sensitivity. Although cryptochrome photochemistry and physiological response characteristics vary throughout the biological Kingdom, and indeed in some cases cryptochrome signaling appears not even to be sensitive to light, it is hoped that this review will help provide a blueprint on how to link *in vitro* mechanisms and redox properties of Crys with an actual physiological responses *in vivo*.

Future perspectives for this field are continuously opening up and will include new ways to manipulate Crys both *in vivo* and *in vitro*, identification of novel Cry variants, and novel applications for Crys in agriculture and in synthetic biology. All these perspectives benefit from an understanding how light signals are transduced into a meaningful physiological response. Additional prospects include design of targeted strategies for manipulation of endogenous Crys in humans to produce ROS (Sherrard et al., 2018), leading to novel therapies such as in the treatment of inflammation and infectious disease (Ronniger et al., 2022).

In combination with suitable lighting protocols, this new technology of ‘magnetogenetics’ could increase the optogenetic toolkit as well as providing fundamental insights on how electromagnetic fields impact on living systems.

## Author contributions

BA: Writing – original draft, Writing – review & editing. JB: Writing – review & editing. SB: Writing – review & editing. NJ: Writing – review & editing. MP: Writing – review & editing. ME: Writing – review & editing. DE: Writing – review & editing. SM: Writing – review & editing. SW: Writing – original draft, Writing – review & editing. AH: Writing – review & editing. KB: Writing – original draft, Writing – review & editing. SK: Writing – original draft, Writing – review & editing. JL: Funding acquisition, Writing – review & editing. MA: Conceptualization, Funding acquisition, Supervision, Validation, Writing – original draft, Writing – review & editing.
